# Development and content validation of a measure to assess evidence-informed decision-making competence in public health nursing

**DOI:** 10.1371/journal.pone.0248330

**Published:** 2021-03-10

**Authors:** Emily Belita, Jennifer Yost, Janet E. Squires, Rebecca Ganann, Maureen Dobbins

**Affiliations:** 1 School of Nursing, McMaster University, Hamilton, Ontario, Canada; 2 M. Louise Fitzpatrick College of Nursing, Villanova University, Villanova, Pennsylvania, United States of America; 3 School of Nursing/École des Sciences Infirmières, University of Ottawa/Université d’Ottawa, Ottawa, Ontario, Canada; 4 National Collaborating Centre for Methods and Tools, McMaster University, Hamilton, Ontario, Canada; Georgia Southern University, UNITED STATES

## Abstract

There are professional expectations for public health nurses to develop competencies in evidence-informed decision-making (EIDM) due to its potential for improved client outcomes. Robust tools to assess EIDM competence can encourage increased EIDM engagement and uptake. This study aimed to develop and validate the content of a measure to assess EIDM competence among public health nurses. A four-stage process, based on measure development principles and the Standards for Educational and Psychological Testing, was used to develop and refine items for a new EIDM competence measure: a) content coverage assessment of existing measures; b) identification of existing measures for use and development of items; c) validity assessment based on content; d) validity assessment based on response process. An EIDM competence measurement tool consisting of EIDM knowledge, skills, attitudes/beliefs, and behaviour items was developed using conceptual literature and existing measures (Evidence-Based Practice Competency Tool and Evidence-Based Practice Beliefs Scale) to address limitations of existing EIDM tools identified from the content coverage assessment. Item content validity index ratings ranged from 0.64–1.00. Qualitative themes from validity assessment based on content and response process included word changes to improve clarity, reducing item redundancy, separating multi-component items, and ensuring items reflect nursing role expectations. Upon determining its reliability and validity, there is potential for the EIDM competence measure to be used in: public health nursing practice to identify competence gaps and strengths to facilitate professional development activities; in research to support development of strategies to build EIDM capacity; and for curriculum planning and development across nursing education programs.

## Introduction

Evidence-informed decision-making (EIDM) is important to the delivery of effective and cost-efficient public health services [1]. EIDM is the integration of the best available research evidence along with consideration of local context, community and political preferences, professional expertise, and resources in public health decision-making [2]. The model for guiding the process of EIDM consists of seven steps: 1) define (clearly defining a public health practice question); 2) search (searching for research evidence); 3) appraise (critically appraising research evidence); 4) synthesize (interpreting and forming practice recommendations based on literature); 5) adapt (adapting research to local context); 6) implement (planning how to implement adapted evidence); and 7) evaluate (evaluating implementation) [2]. Because of its impact on public health outcomes and resources, public health nurses are expected to support EIDM implementation in their practice. For example, EIDM expectations are articulated in community health nursing standards [3], in standards for mandatory public health programs and services [4], and in national public health core competency documents [5].

With EIDM expectations established, the assessment of nurses’ competence in EIDM requires attention. Nursing competence is defined as the quality of a nurse’s ability to use the four attributes of knowledge, skills, attitudes/beliefs, and behaviours in performing a nursing role to an expected standard [6–10]. In the context of EIDM, knowledge is defined as an understanding of the theoretical and practical principles or steps of EIDM (e.g., knowing the hierarchy of research evidence or different tools that can be used to critically appraise evidence) [11–13]. EIDM skills are defined as applying knowledge in the performance of EIDM tasks in a practical setting such as in using a clinical case scenario (e.g., having the ability to conduct a literature search of online databases to address a clinical problem) [12–14]. EIDM attitudes and beliefs represent perceptions or beliefs about and importance of EIDM (e.g., believing that EIDM is associated with positive outcomes) [13, 14] and EIDM behaviours consist of enacting EIDM steps in real-world health care settings (e.g., identifying a gap in client care and defining a clinical problem by establishing an answerable research question) [13–15]. Competence assessment in nursing practice is a critical endeavour because it supports workforce development by identifying professional development needs which can then be addressed through capacity development [16]. This highlights the importance of conceptually sound and robust tools to support competence assessment. However, there are limitations among existing measures that assess EIDM competence attributes.

A systematic review of 35 EIDM competence attribute measures (i.e., knowledge, skills, attitudes/beliefs, and behaviours) determined that the majority of measures assessed only one competence attribute, with only three measures assessing all four attributes [17]. These three measures include the Evidence-Based Practice Questionnaire (EBPQ) [18], the School Nursing Evidence-Based Practice Questionnaire [19], and a self-developed measure by Chiu et al. [20]. While these three measures address all four competence attributes, there are limitations among them. Most importantly, the measures are based on an incomplete conceptualization of EIDM competence. Instead of assessing the quality of attributes, a critical component of competence [7], in some instances, there is a focus on rating items based on agreement or frequency of completing EIDM activities. This makes it challenging to distinguish the ‘quality’ of EIDM knowledge, skill, or behaviours of nurses. As well, for two of these measures, the School Nursing Evidence-Based Practice Questionnaire [19] and a self-developed measure by Chiu et al. [20], behaviour items are narrowly focused on use of online databases, and do not capture the breadth of all EIDM steps. Lastly, in one measure, knowledge and skills attributes are combined into one subscale, when literature identifies them as conceptually different [13]. While this is a recently published systematic review, results from other psychometric systematic reviews have demonstrated similar limitations among existing EIDM measures.

Leung, Trevena, and Waters [12] conducted a systematic review of 59 studies and 24 distinct instruments assessing EIDM knowledge, skills, and attitudes used among nurses and midwives. Of the 24 tools assessed, Leung et al. [12] reported that only one, the Evidence-Based Practice Questionnaire (EBPQ) [18], was deemed as having adequate validity. However, Leung et al. [12] identify important limitations with the EBPQ which assesses knowledge/skills, attitudes, and practice of EIDM (behaviours). Leung et al. [12] note a lack of breadth and clarity with respect to certain items within the EBPQ. For example, in the knowledge and skills subscale, participants are asked to respond to items such as “Research skills”. This item appears out of scope and relates more to the production of research rather than the use of it in decision-making.

In their seminal psychometric systematic review, Shaneyfelt et al. [15] summarized data across 115 studies and 104 distinct EIDM tools used among medical students, trainees, physicians, and other healthcare professionals. Findings from this review also show an emphasis on only one or two competence attributes; 57% of the tools assessed only EIDM skills, 38% assessed knowledge and behaviours, and 26% assessed only attitudes [15]. Shaneyfelt et al. [15] also note a large proportion of the tools focused primarily on the ‘search’ and ‘appraise’ steps, with less emphasis on other EIDM steps. In addition, majority of measures failed to address the EIDM step of ‘adapting evidence to local context’ in that they only assess ability to integrate research evidence in decision-making, neglecting the integration of clinical/local context of patient/client preferences. Authors of psychometric systematic reviews in the field of allied health have also reported findings highlighting conceptual limitations among EIDM tools. Fernandez-Dominguez et al. [21] concluded from their systematic review of 24 tools assessing EIDM among physiotherapists that there is a dearth of well-developed and conceptually robust measures. Authors cite a primary limitation as being a lack of established theoretical or operational definitions of the EIDM constructs under measurement to guide content development, which may contribute to conceptual ambiguity or irrelevance within an instrument. Buchanan, Siegfried, and Jelsma [14] also support this finding in a systematic review of 34 instruments measuring EIDM knowledge, skills, attitudes, and behaviours among occupational therapists. Buchanan et al. [14] note major deficiencies related to tool developers failing to establish concrete definitions of EIDM constructs under measure.

Given that conceptual limitations of existing EIDM measures have persisted over the past 15 years, such that they do not satisfy a comprehensive understanding and assessment of EIDM competence, there was a need for development of a tool that reflects a holistic assessment of EIDM competence.

## Methods

**Fig 1:**
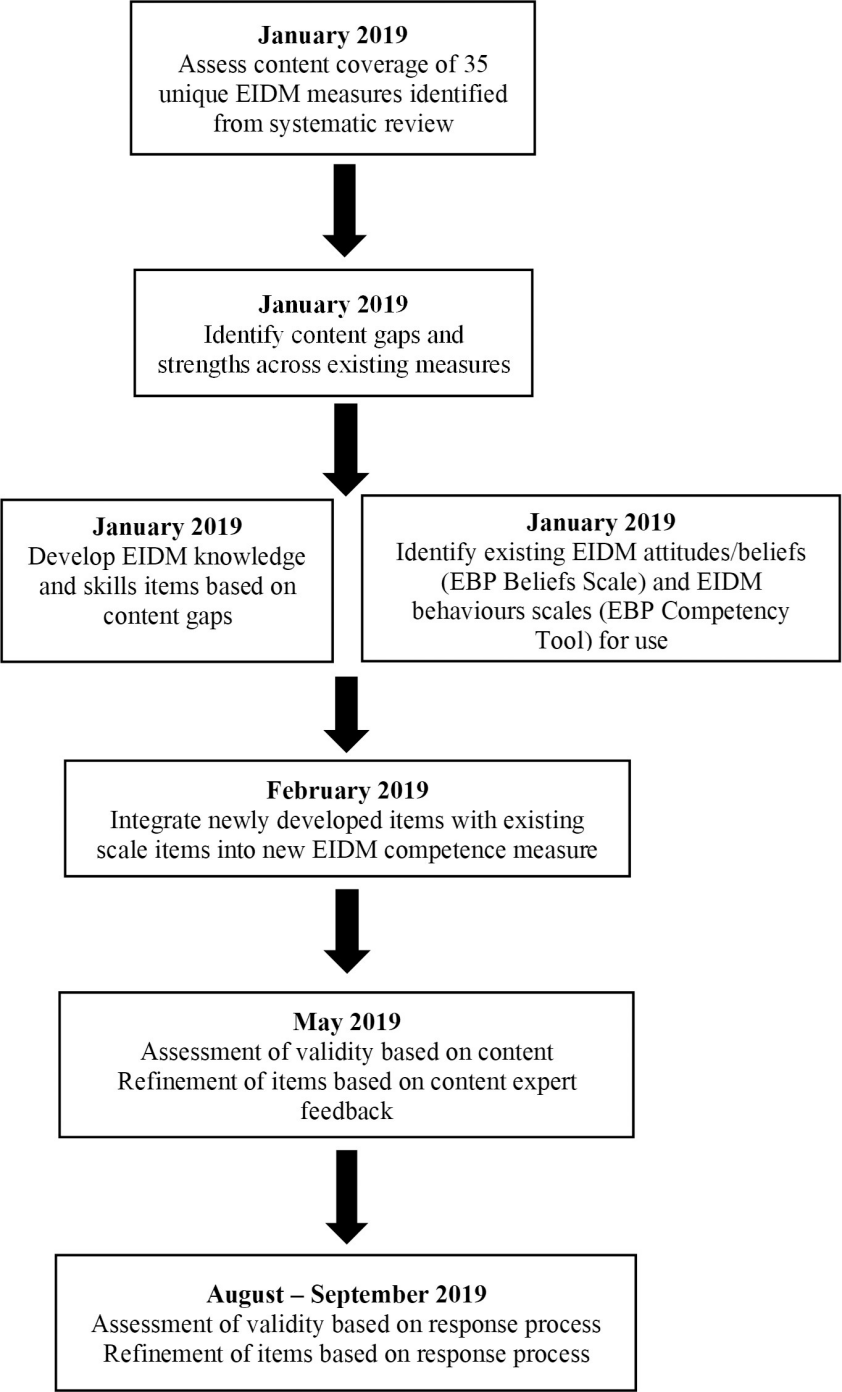
Development process of EIDM competence measure.

Ethics approval for this research study was granted from the Hamilton Integrated Research Ethics Board (HiREB), project #5238. A four-stage process based on measure development principles recommended by Streiner et al. [22] and the Standards for Educational and Psychological Testing [23] was used to develop and refine items for a new EIDM competence measure: a) content coverage assessment; b) identification of existing scales for use and development of items; c) validity assessment based on content; d) validity assessment based on response process. See Fig 1 for an overview of the development and refinement process.

The Standards for Educational and Psychological Testing have a long history of existence extending across the past 60 years with updated revisions to reflect an evolution of validity theory [24]. The standards have been developed using consensus discussions among a panel of psychometric and testing experts from three prominent organizations (American Psychological Association, the American Educational Research Association, and the National Council on Measurement in Education [25]. In addition, rounds of feedback have been solicited from additional experts across these three organizations, testing companies, professional organizations, credentialing organizations, and academic and research institutions to inform ongoing revisions of the standards. The purpose of these standards is to provide criteria in developing and evaluating tests with a specific focus on assessing validity evidence of test scores [23]. The Standards for Educational and Psychological Testing [23] have been used previously to guide the development and validation of other instruments such as a measure to assess conceptual research utilization among healthcare workers [26] and a tool to assess performance (problem solving, use of information, group process, professionalism) in a problem-based learning environment among a sample of medical students [27].

### Content coverage assessment

According to Streiner et al. [22] a first step in devising items for a tool is to review previously completed work to determine if existing scales are adequate and comprehensively cover the construct domains being measured. To this end, a systematic review was undertaken to conduct a content coverage assessment which included 35 unique measures of EIDM competence attributes (i.e., knowledge, skills, attitudes/beliefs, behaviours) [17]. Assessing content coverage using a matrix determines how representative items are across content domains for a concept under measure [22]. To assess content coverage of existing measures, one reviewer (EB) first extracted data on EIDM steps addressed by each measure, which was then checked for accuracy by a second reviewer (TB/TD). Individual items were extracted, where available, from each measure and categorized according to each of the seven steps of EIDM. Reviewers also extracted data on response type for each measure to determine how items were assessed. Measures were categorized according to objective (multiple choice, short answer/open text) or self-report (agreement level, frequency, or quality rating) response type. To assess content coverage of measures addressing EIDM attitudes/beliefs, reviewers extracted available items from each measure and categorized them according to three domains identified from the literature: organizational, personal characteristics, and general beliefs about EIDM [13, 28, 29]. For all attributes, the total number of measures addressing each of the EIDM steps or domains were reported.

### Identification of existing scales for use and development of items

The content coverage assessment identified conceptual gaps among EIDM knowledge and skills measures. Streiner et al. [22] identify that existing research or literature can serve as sources for developing new items. As such, to address these gaps, EIDM knowledge and skill items were developed guided by existing EIDM literature [2, 13, 30–33]. Among the EIDM attitudes and behaviour measures, two measures, the EBP Beliefs Scale [34] and the EBP Competency Tool [35] demonstrated content comprehensiveness; that is, all EIDM content domains were addressed and items had sufficient/specific detail. The original developer (Dr. Bernadette Melnyk) of these tools, provided permission for their use, integration, and modification of specific items. Newly developed EIDM knowledge and skills items and existing EIDM attitudes and behaviour scale items from the EBP Beliefs Scale [34] and the EBP Competency tool [35] were integrated into a new EIDM competence measure and assessed for validity based on content and response process.

### Validity assessment based on content

#### Recruitment and sample

According to the Standards for Educational and Psychological Testing, assessment of validity based on content is defined as “an analysis of the relationship between the content of a test and the construct it is intended to measure” [23]. Assessment of validity based on content can be facilitated through the use of a content expert panel who individually judge the relevance of each proposed item [22, 23]. A purposive sample of international experts in public health and/or EIDM were recruited to participate in the study via email. A list of 17 EIDM experts was generated through knowledge of co-investigators, knowledge of those cited frequently in the related literature, and from the participant list of an EIDM public health conference (2018 FUSE International Conference on Knowledge Exchange in Public Health). This sample size exceeds the minimum recommendation of five experts to assess content validity of a measure [36]. Experts who confirmed interest in participating via email were each sent a unique link to an online consent form and anonymous survey via the platform LimeSurvey.

#### Data collection

Data were collected at one time point in May 2019. In an online survey consisting of 63 items across the competence attribute subscales of EIDM knowledge, skills, attitudes/beliefs, and behaviours, content experts were asked to rate the relevance of each item to the competence attribute under which it was categorized according to a 4-point scale: 1 –not relevant, 2 –unable to assess relevance without item revision or item is in need of revision, 3 –relevant but needs minor alterations, 4 –very relevant and succinct [37]. Experts were also provided an opportunity to write open-text comments for each proposed item.

#### Data analysis

A content validity index (CVI) was calculated at the item (i.e. I-CVI) and scale level from content experts’ relevance ratings [38]. The item CVI is the proportion of experts rating each item as a “3” or “4” [37]. The scale CVI (i.e. S-CVI/Ave) is calculated as the average proportion of items rated a ‘‘3’ or “4” across all judges [38]. Qualitative comments were analyzed using thematic analysis [39].

#### Item refinement

Results were discussed among co-investigators and consensus was used to make decisions regarding item deletions, modifications, and additions with the following guiding principles which were all equally weighted in this process: a) CVI minimum guideline of ≥0.78 for acceptability [37]; b) recommendations from qualitative comments; c) applicability of use in a public health practice setting.

### Validity assessment based on response process

#### Sample and recruitment

Based on the Standards for Educational and Psychological Testing the response process assessment involves an understanding about the thought processes used in responding to scale items and its consistency with the construct being studied [23]. The primary method to assess response process validity is through the use of cognitive interviewing [22, 40]. Streiner et al. [22] recommend that such pilot test interviews of new instruments continue until saturation is reached (i.e., no new concerns are identified), which commonly occurs with a minimum of eight participants. For this study, a convenience sample of nine Public Health Nurses (PHNs) were recruited across two public health units. Criteria for inclusion was that participants had to have the professional designation of registered nurse (RN) and work in any frontline or administrative role in the public health unit. A primary contact at each public health unit disseminated an email to nurses working in any position or role across the health unit to determine interest in study participation.

#### Data collection

The first author (EB) conducted individual 30 minute semi-structured interviews via telephone to test the refined EIDM competence measure for validity evidence based on response process [22]. Participants received an email with a web link to a consent form and anonymous online survey with items from the new measure. Upon participants providing consent, phone interviews were recorded via Skype. After answering 1–3 items at a time, participants were asked semi-structured questions to explore comprehension and ease or difficulty after answering each item [41]. Detailed interview notes were also taken to supplement audio recordings.

#### Data analysis

The ‘interviewer text summary’ model of analysis was used to analyze data, consisting of a “description of dominant themes, conclusions, and problems that are evidenced within a set of aggregated interviewer notes” [42]. Detailed interview notes and digitally recorded interviews were reviewed to identify common themes across participant data. Items were refined based on identified themes and through consensus in discussions among co-investigators.

## Results

### Content coverage assessment

Across 35 measures, items for 28 of them were obtained. Overall, across EIDM knowledge, skill, and behaviour measures, there was a large content emphasis on the ‘search’ and ‘appraise’ steps of EIDM and much less emphasis on the steps of ‘synthesize’ and ‘adapt’ (see Table 1). Across measures, certain individual items were vague, lacking specificity (e.g., I know how to find evidence for practice) [43], or broad in nature (e.g., My knowledge of the application of EBP principles is sufficient) [20]. None of the measures assessing EIDM knowledge or skills assessed all EIDM steps. Only one of the measures assessing EIDM behaviour, the EBP Competency Tool [35] addressed all EIDM steps (see S1 Table). Across EIDM attitudes/beliefs measures, content focused more on general beliefs about EIDM (e.g., I value EBP) [44] as compared to individual/personal or organizational factors. Only one measure, the EBP Beliefs Scale [34], comprehensively addressed all three domains (see S2 Table).

**Table 1:** Number (%) of EIDM knowledge, skills, behaviours, and attitude EIDM measures that address each of the EIDM steps.

EIDM competence attribute addressed	Number of measures addressing EIDM steps								
	General	Define	Search	Appraise	Synthesize	Adapt		Implement	Evaluate
**Knowledge (n = 19 measures)**^**a**^	11 (57.9%)	5 (26.3%)	7 (26.3%)	6 (31.6%)	2 (10.5%)	0		3 (15.8%)	1 (5.3%)
**Skills (n = 15 measures)**^**a**^	2 (13.3%)	6 (40%)	10 (66.7%)	9 (60%)	1 (6.7%)	2 (13.3%)		5 (33.3%)	4 (26.7%)
**Behaviours (n = 13 measures)**^**a**^	4 (30.8%)	8 (61.5%)	11 (84.6%)	10 (76.9%)	5 (38.5%)	5 (38.5%)		8 (61.5%)	7 (53.8%)
**Attitudes (n = 17 measures)**^**a**^	**Number of measures addressing EIDM attitudes/beliefs steps**								
	**General beliefs about EIDM**			**Individual/personal factors**			**Organizational factors**		
	14 (82.4%)			6 (35.3%)			5 (29.4%)		

^a^Measures in each category identified in S1 Table.

Based on identified content gaps, lack of specificity, and vagueness in existing items, new self-report items were generated for EIDM knowledge (19 items) and EIDM skills (15 items) subscales. Response scales assessing quality of EIDM knowledge and skill acquisition were also developed using psychometric principles [22] and conceptual literature on competence [7]. New items were integrated with items from the EBP Competency Tool (n = 13) [35] and EBP Beliefs Scale (n = 16) [34], which comprehensively addressed EIDM behaviours and attitudes/beliefs respectively. In total, 63 items were proposed to assess EIDM competence through assessment of knowledge, skills, attitudes/beliefs, and behaviours.

With respect to response type, the majority of tools were self-report compared to more objective measures (See S3 Table). Among the EIDM knowledge measures (n = 19), six were objective (31.6%), while 10 (52.6%) were self-report, and response type data could not be obtained for three measures. Objective EIDM knowledge measures were equally balanced between multiple choice (n = 2), open text/short answer (n = 2), and a combination of multiple choice and short answer (n = 2). Self-report EIDM knowledge measures most frequently had agreement level (n = 5) or quality rating (n = 5) scales. Of the EIDM skills measures (n = 15), a greater number were self-report (n = 10) compared to objective (n = 2), and data could not be retrieved for three measures. Response scales types differed across self-report EIDM skills measures: agreement level (n = 3), quality rating (n = 6); and confidence level (n = 2). All EIDM behaviour measures were of a self-report nature (n = 13). Majority of the EIDM behaviour measures used frequency response scales (n = 8) compared to employing an agreement level scale (n = 2), a quality rating scale (n = 2), or a confidence scale (n = 2). All of the EIDM attitudes/beliefs measures (n = 17) used an agreement level response scale.

### Assessment of validity evidence based on content

Of the 17 international EIDM experts that were contacted, 11 (65%) participated in the online survey (5 from Europe, 2 from the United States, 4 from Canada) to assess validity based on content of the new measure (63 items). Across the entire measure, item CVIs ranged from 0.64–1.00. Ranges of I-CVIs were similar across subscales: EIDM knowledge (0.72–1.00); EIDM skills (0.72–1.00); EIDM attitudes/beliefs (0.64–0.91); and EIDM behaviours (0.72–0.91) (see S4 Table for CVIs of individual items). Scale-CVIs varied across subscales: knowledge (0.88); skills (0.88); attitudes/beliefs (0.79); and behaviours (0.87). Across subscales, qualitative comments centred on four main themes. First, content experts recommended specific word changes to items to increase clarity:

*“The 6S hierarchy is a very specific item–are all PHNs trained on this particular (i*.*e*., *Haynes’) version*?*–would it be sufficient (or more appropriate) to talk about an evidence pyramid/hierarchy (i*.*e*., *mention the concept of the hierarchy rather than a specific representation of it)*?*”* (feedback for EIDM knowledge item)

Second, experts also identified points of redundancy across items:

*“Dissemination of best practice is likely to be part of the implementation step mentioned in item 10*. *I would reduce this overlap and false dichotomy by using item 10 instead*.*”* (feedback for EIDM behaviours item)

Third, qualitative data in some instances, suggested combining certain items or separating double-barrelled items (i.e., items that ask two or more questions simultaneously):

*“Other sections want respondent to specify/respond to this question about critical appraisal according to different designs (multiple questions) how consistent is it to lump them all into one question here (though maybe that would be a better strategy for the earlier sections*, *to combine into a general question)”* (feedback for EIDM behaviour item)

*“These steps are complex processes*. *Do you want questions for each one*?*”* (feedback for EIDM knowledge item)

And fourth, comments conveyed that some items were not reflective of EIDM expectations for nurses:

*“This is borderline to conducting research… questionable as whether part of EBP/EIDM–will every practitioner be able to do this*?*”* (feedback on EIDM behaviour item)

After considering CVIs, qualitative feedback, and feasibility for use in public health practice settings across the whole measure, 28 items were deleted, 23 were modified, 5 items were added, and 12 were kept in their original form. See Table 2 for data according to each subscale. After these revisions, a total of 40 items were proposed with varying numbers across subscales: EIDM knowledge (11 items); EIDM skills (10 items); EIDM attitudes/beliefs (7 items); and EIDM behaviours (12 items). These modified items then underwent an assessment of validity based on response process in the next phase of measure development.

**Table 2:** Deleted and modified items following content validity assessment.

Subscale	Number of original items	Number of deleted items	Number of modified items	Number of new items added	Number of items kept in original form	Total # after revisions
**Knowledge**	19	8	4	0	**7**	**11**
**Skills**	15	7	5	2	3	**10**
**Attitude/Beliefs**	16	9	5	0	2	**7**
**Behaviours**	13	4	9	3	0	**12**

### Assessment of validity based on response process

Nine registered nurses in frontline (n = 7) or supervisory roles (n = 2) from two public health units, participated in the assessment of validity based on response process. No items were deleted or added following this assessment. Eight items were modified across all subscales of knowledge (n = 3 items), skills (n = 1 item), attitudes/beliefs (n = 3 items), and behaviours (n = 1 item). Across all modified items, minor revisions followed three main categories to increase clarity: removing words; adding examples; or re-ordering words.

One theme that emerged specific to the knowledge items was that while participants generally felt items were clear and straightforward, some items included terms that required further explanation (e.g., knowledge of what is involved in the ‘search’ step of EIDM). Participants identified a need for information to help clarify terms that denoted specific steps in EIDM. Three participants suggested use of an information box that hovers over and provides brief definitions of broad EIDM terms (e.g., synthesize, adapt).

With respect to the behaviour items, the majority of participants felt that the stem of each item needed further clarity by adding “I” to the beginning of the statement (i.e., ‘participates in the formulation of public health practice questions’ versus ‘I participate in the formulation of public health practice questions). As well, the response scale for behaviour items was changed from a 4-point to a 7-point Likert scale, based on participant feedback to improve scale consistency, since the other subscales consisted of a 7-point response scale. See Table 3 for the final 40-item scale.

**Table 3:** 40-item EIDM competence measure.

**EIDM Knowledge Items (1 = poor to 7 = excellent)**
1. Knowledge of what is involved in the ‘define’ step of EIDM.
2. Knowledge of what is involved in the ‘search’ step of EIDM.
3. Knowledge about the different levels of evidence when searching for research evidence (e.g., single studies, systematic reviews, summaries)
4. Knowledge that online databases exist which house publications of individual research studies (e.g., PubMed, CINAHL).
5. Knowledge that online databases exist which house pre-appraised, synthesized research evidence (e.g., Health Evidence, ACCESSSS)
6. Knowledge of what is involved in the ‘appraise’ step of EIDM.
7. Knowledge that critical appraisal tools exist to assess the quality of research evidence (e.g., AGREE II tool, CASP).
8. Knowledge of what is involved in the ‘synthesize’ step of EIDM.
9. Knowledge of what is involved in the ‘adapt’ step of EIDM.
10. Knowledge of what is involved in the ‘implement’ step of EIDM.
11. Knowledge of what is involved in the ‘evaluate’ step of EIDM.
**EIDM Skills Items (1 = beginner to 7 = expert)**
1. Ability to develop an answerable practice question.
2. Ability to develop an appropriate strategy to search for research evidence.
3. Ability to use online databases that house publications of individual research studies (e.g., CINAHL).
4. Ability to use online databases that house pre-appraised, synthesized research evidence (e.g., Health Evidence).
5. Ability to use critical appraisal tools to appraise the quality of research evidence (e.g., AGREE II tool, CASP)
6. Ability to assess the applicability of research evidence to the local public health context.
7. Ability to conduct an assessment of barriers and facilitators (related to resources, organization, evidence/guideline, clients’ preferences/values) when implementing a practice change.
8. Ability to conduct a stakeholder analysis (i.e. collecting and analyzing information on stakeholders’ importance and influence) when implementing a practice change.
9. Ability to develop an action plan to implement an evidence-informed practice change.
10. Ability to participate in the development of evaluation indicators to assess outcomes of evidence-informed decisions or practice changes.
**EIDM Attitudes/Beliefs Items (1 = strongly disagree to 7 = strongly agree)**
1. I believe that I can implement EIDM in a time efficient way.
2. I believe that I can engage others in implementing strategies to address barriers (e.g., personal, organizational, community) when implementing EIDM.
3. I believe that evaluating outcomes of an evidence-informed decision/practice change is an important component of EIDM.
4. I believe that implementing EIDM can improve the services and programs delivered to clients (e.g., communities, individuals, families).
5. I believe that critically appraising evidence is an important step in the EIDM process.
6. I believe that the use of high- quality evidence-informed guidelines (e.g., clinical practice guidelines) can improve public health practice and policy.
7. I believe EIDM is difficult. (reverse scored)
**EIDM Behaviour Items (1 = not competent to 7 = highly competent)**
1. I question public health practices for the purpose of improving the quality of care/service delivery.
2. I describe public health practice issues using client assessment data (i.e., community, individuals, families, populations).
3. I participate in the formulation of public health practice questions.
4. I search for research evidence to answer public health practice questions.
5. I participate in the critical appraisal of individual research studies to determine their strength and applicability to public health practice.
6. I participate in the critical appraisal of synthesized evidence (such as clinical practice guidelines, evidence-based policies and procedures, and evidence syntheses).
7. I participate in the synthesis and interpretation of a body of research evidence gathered to formulate recommendations for public health practice.
8. I integrate evidence gathered from public health expertise, client/community preferences, and local context with research evidence to plan evidence-informed practice changes.
9. I participate in the assessment of barriers and facilitators (related to resources, organization, evidence/guidelines, clients’ preferences/values) when implementing a practice change.
10. I participate in the process of stakeholder analyses (i.e., collecting and analyzing information on stakeholders’ importance and influence) when implementing a practice change.
11. I participate in the development of an action plan to implement a practice change.
12. I participate in evaluating outcomes of evidence-informed decisions or practice changes.

## Discussion

### Content coverage assessment

This study reports on the development and first phase of validation for a self-report EIDM competence measure used in public health nursing. The first step used in developing an initial item pool was content coverage assessment.

Results from this content coverage assessment showed notable trends. Items across measures more frequently addressed the EIDM steps of ‘search’ and ‘appraise’. Steps that appear later in the EIDM process (i.e., synthesize, adapt) were less often addressed across measures. This emphasis on searching for, retrieving, and critically appraising research evidence was also demonstrated in a systematic review of 104 EIDM measures used by physicians and trainees [15]. Shaneyfelt and colleagues [15] reported that measures assessing EIDM skills focused heavily on appraising quality of research evidence and searching specific online databases. Perhaps this focus on assessment of the first steps of EIDM is largely influenced by the content of current educational interventions that aim to develop EIDM knowledge, skills, and behaviours. In a systematic review of eight studies on educational interventions that promoted learning of EIDM among nurses, learning content was analyzed, showing a primary sub-theme of searching for and evaluating evidence [45]. This was similarly found in another systematic review of training interventions to develop EIDM knowledge and skills among healthcare professionals [46]. Phillips et al. [46] reported that among the 61 intervention studies included in their review, the most frequently addressed steps were related to appraise (n = 46; 75%) and search (n = 38; 62%). With a concentration on the initial steps of the EIDM process, there is a need to expand the breadth of EIDM competence assessment and content in educational interventions to support a holistic development of EIDM competence [47]. A unique contribution of our proposed EIDM competence measure is that it encompasses items that specifically assess all steps in the EIDM process across knowledge, skills, and behaviour subscales.

Content coverage assessment also determined that existing self-report EIDM measures which assess knowledge, skills, attitudes/beliefs, and behaviours have response scales that do not assess the quality of a competence attribute, but rather use agreement or frequency scales [18–20]. A conceptual limitation of this approach is that it reduces EIDM to completion of tasks, rather than focusing on knowledge level, and how well a skill or behaviour is being performed [9]. Integrated in this new EIDM competence measure are response scales (e.g., beginner to expert; poor to excellent) that reflect quality or one’s ability to perform an EIDM task, a critical component of ongoing competence assessment for workforce development [7, 10].

### Validity based on content

With respect to validity based on content for our measure, item level content validity indices (CVI) were computed [38]. Among existing literature, in a systematic review of 35 EIDM measures [17], CVIs were used to confirm validity based on content for only four measures: the Quick VIK (Values, Implementation, Knowledge) survey [44]; the Knowledge and Skills in Evidence-Based Nursing Tool [48]; Modified Stevens EBP Readiness Inventory [49]; and a self-developed tool by Bostrom et al. [50]. For the four measures with computed CVIs identified above, the majority of original items had CVIs between 0.80–1.0, indicating acceptable content validity. In comparison, CVIs for initial items in our proposed EIDM competence measure ranged from 0.64–1.0. Most of the low CVI values were linked to items in the attitudes/beliefs subscale, an attribute covered in only one of the four existing EIDM measures reporting CVIs [44]. Less agreement in relevance ratings of EIDM attitudes/beliefs items may be attributed to the subjective nature of this domain compared to greater objectivity surrounding competence indicators for knowledge, skills, and behaviours.

Supplemental to CVI results, qualitative results played a critical role in revisions to the measure. Expert feedback informed, deletions and wording modifications to remove technical terms and simplify multicomponent items. This feedback and measure changes are supported by Streiner and colleagues’ [22] recommendations when selecting or devising items. To improve interpretability of items, Streiner and colleagues stress the importance of pre-testing prior to the use of jargon terms. As well, to decrease cognitive load, it is suggested to separate double-barrelled questions into multiple items instead [22]. Another major qualitative theme that surfaced was ensuring items were congruent with realistic EIDM expectations for nurses. Developing items that accurately reflect EIDM expectations specific to nurses is a valid consideration given there are differences with respect to varying degrees of exposure to EIDM, differing levels of EIDM knowledge and skills, and receptiveness to the EIDM process across disciplines [11].

### Validity based on response process

Validity based on response process was assessed by conducting interviews with nine nurses, in frontline and supervisory roles. Having these two perspectives was beneficial, given that both represent the public health end users for this measure. A strength of our study is that response process assessment demonstrated participants had strong comprehension of the items, with minor word changes suggested to improve clarity on eight items. This validates the extensive work done prior to modify, delete, and develop new items based on results from the assessment of validity based on content.

### Limitations

While this study makes a unique contribution to the EIDM measurement nursing literature, there are limitations to note. First, for some of the measures assessed for content coverage, specific items could not be retrieved despite efforts to contact original developers. However, given that items for only seven of 35 measures could not be obtained, it is unlikely that such a small number would substantially impact results. Even across the 28 measures of which content coverage was assessed, prominent and consistent themes emerged. Second, a convenience sample was used for the response process assessment with potential to bias results. Those who agreed to participate in this stage of the study may already have a strong interest in EIDM, which could skew their comprehension or feedback about the measure’s items. However, in selecting the two public health units from which this sample was obtained, we selected one health unit that was immersed in EIDM work for many years along with a second health unit which was in the beginning stages of conducting EIDM work. This was strategically done to capture diverse perspectives of individuals with differing exposure to EIDM and varied levels of EIDM knowledge, skills, attitudes/beliefs, and behaviours.

### Future areas of research

Regarding future areas of research, additional psychometric assessment of the new self-report EIDM competence measure is currently underway via a pilot project with 16 Ontario public health units. In this pilot project, acceptability, validity, and reliability evidence is being assessed with an extended sample of nurses working in public health in various roles via an online survey. Acceptability testing will include assessing completion time and missing data rates. Additional validity testing will consist of assessing the internal structure of the measure via exploratory factor analysis and determining the measure’s relationship to other variables (e.g., education level, role, EIDM project involvement). And finally, the internal consistency of the measure will be evaluated to assess its reliability. While this measure was developed with the nursing role in mind, there is also potential to assess its psychometric performance in other professional groups of the public health workforce for use in real-world practice.

## Conclusions

A rigorous process was used to develop and validate the content of a proposed EIDM competence measure for use among public health nurses. Validity evidence with respect to content and response process was assessed and results were used to modify, delete, and add new items to ensure content relevance and clarity.

This new EIDM competence measure has great potential to impact nursing practice, education, and research. Specific EIDM competence indicators can be integrated into performance review processes to support public health nurses in identifying learning needs and developing tailored learning plans related to EIDM. Organizations may also use these indicators for workforce planning and management by articulating EIDM roles and responsibilities for public health nurses [16, 51]. In nursing research, having a standardized EIDM competence measure to help identify workforce gaps is a critical first step in developing targeted interventions to address specific EIDM competencies or overall EIDM competence. There also is great potential to apply this understanding about EIDM competence to curriculum planning and development in undergraduate and graduate nursing programs. Methods for assessing EIDM competence can be integrated into nursing curricula with subsequent use of tailored educational strategies based on competence assessment results.

## Supporting information

S1 Table Detailed content coverage chart. (DOCX)

S2 Table Detailed content coverage attitudes/beliefs. (DOCX)

S3 Table Response scales of measures. (DOCX)

S4 Table Content validity indexes. (DOCX)
