# Supplementary material for: Development and content validation of a measure to assess evidence-informed decision-making competence in public health nursing
Source: PLoS One. 2021 Mar 10;16(3):e0248330. doi: 10.1371/journal.pone.0248330 (PMC7946311; doi:10.1371/journal.pone.0248330)
Supplement: S1 Table — (DOCX) [file pone.0248330.s001.docx]

**S1 Table. Detailed content coverage chart per measure.**

| **Tool** | **EIDM Content Coverage** | | | | | | | |
| --- | --- | --- | --- | --- | --- | --- | --- | --- |
|  | **General** | **Define** | **Search** | **Appraise** | **Synthesize** | **Adapt** | **Implement** | **Evaluate** |
| **Knowledge**  (n=19 measures total; n=15 measures with retrieved items) | | | | | | | | |
| **Evidence-Based Practice Questionnaire (EBPQ) (Upton & Upton, 2006)** |  |  |  |  |  |  |  |  |
| **School Nursing Evidence-Based Practice Questionnaire (SN-EBP) (Adams, 2007)** |  |  |  |  |  |  |  |  |
| **Self-developed measure by Chiu et al. (2010)** |  |  |  |  |  |  |  |  |
| **Johns Hopkins Nursing EBP Assessment Survey (Bissett, Cvach, & White, 2016)** |  |  |  |  |  |  |  |  |
| **Persian translated EBP measure (Seyyedrasooli, Zamanzadeh, Valizadeh, & Tadaion, 2012)** | *Unable to retrieve items. | | | | | | | |
| **Self-developed measure by Yip, Mordiffi, Majid, and Ang (2010)** |  |  |  |  |  |  |  |  |
| **Self-developed measure by Chew, Sim, Sim, and Yan (2015)** | *Unable to retrieve items. | | | | | | | |
| **Self-developed EBP measure by B. M. Melnyk et al. (2004)** |  |  |  |  |  |  |  |  |
| **Modified Evidence-Based Nursing Education Questionnaire (EBEQ) (Hellier & Cline, 2016)** |  |  |  |  |  |  |  |  |
| **Quick EBP VIK (Values, Implementation, Knowledge) Survey (Linda Connor, 2017; L. Connor, Paul, McCabe, & Ziniel, 2017)** |  |  |  |  |  |  |  |  |
| **Modified Stevens EBP Readiness Inventory (ERI) (Finnish ERI)**  **(Saunders, Stevens, & Vehvilainen-Julkunen, 2016)** | *Unable to retrieve items. | | | | | | | |
| **Self-developed measure by Gerrish et al. (2011)** |  |  |  |  |  |  |  |  |
| **Knowledge and Skills in Evidence-Based Nursing (KS-EBN) (Gu, Ha, & Kim, 2015)** |  |  |  |  |  |  |  |  |
| **Adapted Fresno Test (Laibhen-Parkes, 2014)** |  |  |  |  |  |  |  |  |
| **Single item measure for EBP knowledge by Skela-Savic, Hvalic-Touzery, and Pesjak (2017)** |  |  |  |  |  |  |  |  |
| **Perceived EBP Knowledge Measure (Thiel & Ghosh, 2008)** |  |  |  |  |  |  |  |  |
| **Evidence-Based Practice Knowledge**  **Assessment in Nursing (EKAN) (Hagedorn Wonder et al., 2017)** |  |  |  |  |  |  |  |  |
| **Knowledge Assessment Test (KAT) (Xie, Zhou, Xu, Ong, & Govindasamy, 2015)** | *Unable to retrieve items. | | | | | | | |
| **Core Knowledge Questionnaire (Toole, Stichler, Ecoff, & Kath, 2013)** |  |  |  |  |  |  |  |  |
| **Total # measures addressing each EIDM knowledge domain** | **11** | **5** | **7** | **6** | **2** | **0** | **3** | **1** |
| **Skills**  (n=15 measures; n=12 measures with items retrieved) | | | | | | | | |
| **EBPQ (Upton & Upton, 2006)** |  |  |  |  |  |  |  |  |
| **SN-EBP (Adams, 2007)** |  |  |  |  |  |  |  |  |
| **Self-developed measure by Chiu et al. (2010)** |  |  |  |  |  |  |  |  |
| **Johns Hopkins Nursing EBP Assessment Survey (Bissett et al., 2016)** |  |  |  |  |  |  |  |  |
| **Persian translated EBP measure (Seyyedrasooli et al., 2012)** | *Unable to retrieve items. | | | | | | | |
| **Self-developed measure by Yip et al. (2010)** |  |  |  |  |  |  |  |  |
| **Self-developed measure by Chew et al. (2015)** | *Unable to retrieve items. | | | | | | | |
| **EBP measure developed by Majid et al.** **(2011) (Adamu & Naidoo, 2015; Farokhzadian, Khajouei, & Ahmadian, 2015)** |  |  |  |  |  |  |  |  |
| **Modified Stevens EBP Readiness Inventory (ERI) (Finnish ERI) (Saunders et al., 2016)** | *Unable to retrieve items. | | | | | | | |
| **Self-developed measure by Gerrish et al.** (2011) |  |  |  |  |  |  |  |  |
| **Knowledge and Skills in Evidence-Based Nursing (KS-EBN) (Gu et al., 2015)** |  | - . |  |  |  |  |  |  |
| **Adapted Fresno Test (Laibhen-Parkes, 2014)** |  |  |  |  |  |  |  |  |
| **Self-developed measure by Gerrish and Clayton (2004)** |  |  |  |  |  |  |  |  |
| **DEBPQ (Gerrish et al., 2007)** |  |  |  |  |  |  |  |  |
| **Information literacy tool (Sim, Jang, & Kim, 2016)** |  |  |  |  |  |  |  |  |
| **Total # measures addressing each EIDM skills domain** | **2** | **6** | **10** | **9** | **1** | **2** | **5** | **4** |
| **Behaviours**  (n=13 measures total and with retrieved items) | | | | | | | | |
| **EBPQ (Upton & Upton, 2006)** |  |  |  |  |  |  |  |  |
| **SN-EBP (Adams, 2007)** |  |  |  |  |  |  |  |  |
| **Self-developed measure by Chiu et al. (2010)** |  |  |  |  |  |  |  |  |
| **Johns Hopkins Nursing EBP Assessment Survey (Bissett et al., 2016)** |  |  |  |  |  |  |  |  |
| **Self-developed EBP measure by Melnyk et al. (2004)** |  |  |  |  |  |  |  |  |
| **Modified Evidence-Based Nursing Education Questionnaire (EBEQ) (Hellier & Cline, 2016)** |  |  |  |  |  |  |  |  |
| **Quick EBP VIK (Values, Implementation, Knowledge) Survey (Linda Connor, 2017; L. Connor et al., 2017)** |  |  |  |  |  |  |  |  |
| **Self-developed measure by Barako, Chege, Wakasiaka, and Omondi (2012)** |  |  |  |  |  |  |  |  |
| **EBP Implementation Scale (B. M. Melnyk, Fineout-Overholt, & Mays, 2008)** |  |  |  |  |  |  |  |  |
| **Self-developed measure by Bostrom, Rudman, Ehrenberg, Gustavsson, and Wallin (2013)** |  |  |  |  |  |  |  |  |
| **Self-developed measure by (Kim et al., 2013)** |  |  |  |  |  |  |  |  |
| **Evidence-Based Practice Confidence Scale (EPIC) (Duffy, Culp, Sand-Jecklin, Stroupe, & Lucke-Wold, 2016; Duffy et al., 2015)** |  |  |  |  |  |  |  |  |
| **EBP Competency Tool (Bernadette Mazurek Melnyk et al., 2018)** |  |  |  |  |  |  |  |  |
| **Total # measures addressing each EIDM behaviours domain** | **4** | **8** | **11** | **10** | **5** | **5** | **8** | **7** |

Adams, S. L. (2007). *Understanding the variables that influence translation of evidence-based practice into school nursing.* (Ph.D.), University of Iowa, Retrieved from <http://search.ebscohost.com/login.aspx?direct=true&db=cin20&AN=109853626&site=ehost-live> (Dissertation/Thesis)

Adamu, A., & Naidoo, J. R. (2015). Exploring the perceptions of registered nurses towards evidence-based practice in a selected general hospital in Nigeria. *Africa Journal of Nursing and Midwifery, 17*(1), 33-46.

Barako, T. D., Chege, M., Wakasiaka, S., & Omondi, L. (2012). Factors influencing application of evidence-based practice among nurses. *African Journal of Midwifery & Women's Health, 6*(2), 71-77.

Bissett, K. M., Cvach, M., & White, K. M. (2016). Improving Competence and Confidence With Evidence-Based Practice Among Nurses: Outcomes of a Quality Improvement Project. *Journal for Nurses in Professional Development, 32*(5), 248-255. doi:<https://dx.doi.org/10.1097/NND.0000000000000293>

Bostrom, A. M., Rudman, A., Ehrenberg, A., Gustavsson, J. P., & Wallin, L. (2013). Factors associated with evidence-based practice among registered nurses in Sweden: a national cross-sectional study. *BMC Health Services Research, 13*, 165. doi:<https://dx.doi.org/10.1186/1472-6963-13-165>

Chew, M. L., Sim, K. H., Sim, Y. F., & Yan, C. C. (2015). Attitudes, skills and knowledge of primary healthcare nurses on the use of evidence-based nursing (EBN) and barriers influencing the use of EBN in the primary healthcare setting. *Annals of the Academy of Medicine Singapore, 1)*, S503.

Chiu, Y. W., Weng, Y. H., Lo, H. L., Shih, Y. H., Hsu, C. C., & Kuo, K. N. (2010). Impact of a nationwide outreach program on the diffusion of evidence-based practice in Taiwan. *International Journal for Quality in Health Care, 22*(5), 430-436. doi:<https://dx.doi.org/10.1093/intqhc/mzq049>

Connor, L. (2017). *Pediatric nurses' knowledge, values, and implementation of evidence-based practice and use of two patient safety goals.* (10269833), University of Massachusetts Boston, Ann Arbor. Retrieved from <http://libaccess.mcmaster.ca/login?url=https://search.proquest.com/docview/1911313970?accountid=12347> <http://sfx.scholarsportal.info/mcmaster?url_ver=Z39.88-2004&rft_val_fmt=info:ofi/fmt:kev:mtx:dissertation&genre=dissertations+%26+theses&sid=ProQ:ProQuest+Dissertations+%26+Theses+A%26I&atitle=&title=Pediatric+Nurses%27+Knowledge%2C+Values%2C+and+Implementation+of+Evidence-Based+Practice+and+Use+of+Two+Patient+Safety+Goals&issn=&date=2017-01-01&volume=&issue=&spage=&au=Connor%2C+Linda&isbn=9781369816976&jtitle=&btitle=&rft_id=info:eric/&rft_id=info:doi/> (Dissertation/Thesis)

Connor, L., Paul, F., McCabe, M., & Ziniel, S. (2017). Measuring Nurses' Value, Implementation, and Knowledge of Evidence-Based Practice: Further Psychometric Testing of the Quick-EBP-VIK Survey. *Worldviews on Evidence-Based Nursing, 14*(1), 10-21. doi:<https://dx.doi.org/10.1111/wvn.12190>

Duffy, J. R., Culp, S., Sand-Jecklin, K., Stroupe, L., & Lucke-Wold, N. (2016). Nurses' Research Capacity, Use of Evidence, and Research Productivity in Acute Care: Year 1 Findings From a Partnership Study. *Journal of Nursing Administration, 46*(1), 12-17. doi:<https://dx.doi.org/10.1097/NNA.0000000000000287>

Duffy, J. R., Culp, S., Yarberry, C., Stroupe, L., Sand-Jecklin, K., & Sparks Coburn, A. (2015). Nurses' research capacity and use of evidence in acute care: baseline findings from a partnership study. *Journal of Nursing Administration, 45*(3), 158-164. doi:<https://dx.doi.org/10.1097/NNA.0000000000000176>

Farokhzadian, J., Khajouei, R., & Ahmadian, L. (2015). Evaluating factors associated with implementing evidence-based practice in nursing. *Journal of Evaluation in Clinical Practice, 21*(6), 1107-1113. doi:<https://dx.doi.org/10.1111/jep.12480>

Gerrish, K., Ashworth, P., Lacey, A., Bailey, J., Cooke, J., Kendall, S., & McNeilly, E. (2007). Factors influencing the development of evidence-based practice: a research tool. *Journal of Advanced Nursing, 57*(3), 328-338. doi:<https://dx.doi.org/10.1111/j.1365-2648.2006.04112.x>

Gerrish, K., & Clayton, J. (2004). Promoting evidence-based practice: an organizational approach. *Journal of Nursing Management, 12*(2), 114-123.

Gerrish, K., Guillaume, L., Kirshbaum, M., McDonnell, A., Tod, A., & Nolan, M. (2011). Factors influencing the contribution of advanced practice nurses to promoting evidence-based practice among front-line nurses: findings from a cross-sectional survey. *Journal of Advanced Nursing, 67*(5), 1079-1090. doi:<https://dx.doi.org/10.1111/j.1365-2648.2010.05560.x>

Gu, M. O., Ha, Y., & Kim, J. (2015). Development and validation of an instrument to assess knowledge and skills of evidence-based nursing. *Journal of Clinical Nursing, 24*(9-10), 1380-1393.

Hagedorn Wonder, A., McNelis, A. M., Spurlock, D. J., Ironside, P. M., Lancaster, S., Davis, C. R., . . . Verwers, N. (2017). Comparison of Nurses' Self-Reported and Objectively Measured Evidence-Based Practice Knowledge. *Journal of Continuing Education in Nursing, 48*(2), 65-70. doi:<https://dx.doi.org/10.3928/00220124-20170119-06>

Hellier, S., & Cline, T. (2016). Factors that affect nurse practitioners' implementation of evidence-based practice. *Journal of the American Association of Nurse Practitioners, 28*(11), 612-621. doi:<https://dx.doi.org/10.1002/2327-6924.12394>

Kim, S. C., Brown, C. E., Ecoff, L., Davidson, J. E., Gallo, A.-M., Klimpel, K., & Wickline, M. A. (2013). Regional Evidence-Based Practice Fellowship Program: Impact on Evidence-Based Practice Implementation and Barriers. *Clinical Nursing Research, 22*(1), 51-69. doi:10.1177/1054773812446063

Laibhen-Parkes, N. (2014). *Web-Based evidence based practice educational intervention to improve EBP competence among BSN-prepared pediatric bedside nurses: A mixed methods pilot study.* (Ph.D.), Mercer University, Retrieved from <http://search.ebscohost.com/login.aspx?direct=true&db=cin20&AN=109753992&site=ehost-live> (Dissertation/Thesis)

Melnyk, B. M., Fineout-Overholt, E., Fischbeck Feinstein, N., Li, H., Small, L., Wilcox, L., & Kraus, R. (2004). Nurses' perceived knowledge, beliefs, skills, and needs regarding evidence-based practice: implications for accelerating the paradigm shift. *Worldviews on Evidence-Based Nursing, 1*(3), 185-193. doi:<https://dx.doi.org/10.1111/j.1524-475X.2004.04024.x>

Melnyk, B. M., Fineout-Overholt, E., & Mays, M. Z. (2008). The evidence-based practice beliefs and implementation scales: psychometric properties of two new instruments. *Worldviews on Evidence-Based Nursing, 5*(4), 208-216. doi:<https://dx.doi.org/10.1111/j.1741-6787.2008.00126.x>

Melnyk, B. M., Gallagher‐Ford, L., Zellefrow, C., Tucker, S., Thomas, B., Sinnott, L. T., & Tan, A. (2018). The First U.S. Study on Nurses’ Evidence‐Based Practice Competencies Indicates Major Deficits That Threaten Healthcare Quality, Safety, and Patient Outcomes. *Worldviews on Evidence-Based Nursing, 15*(1), 16-25. doi:10.1111/wvn.12269

Saunders, H., Stevens, K. R., & Vehvilainen-Julkunen, K. (2016). Nurses' readiness for evidence-based practice at Finnish university hospitals: a national survey. *Journal of Advanced Nursing, 72*(8), 1863-1874. doi:<https://dx.doi.org/10.1111/jan.12963>

Seyyedrasooli, A., Zamanzadeh, V., Valizadeh, L., & Tadaion, F. (2012). Individual Potentials Related to Evidence-Based Nursing among Nurses in Teaching Hospitals Affiliated to Tabriz University of Medical Sciences, Tabriz, Iran. *Journal of Caring Sciences, 1*(2), 93-99. doi:<https://dx.doi.org/10.5681/jcs.2012.014>

Sim, J. Y., Jang, K. S., & Kim, N. Y. (2016). Effects of education programs on evidence-based practice implementation for clinical nurses. *Journal of Continuing Education in Nursing, 47*(8), 363-371. doi:<https://dx.doi.org/10.3928/00220124-20160715-08>

Skela-Savic, B., Hvalic-Touzery, S., & Pesjak, K. (2017). Professional values and competencies as explanatory factors for the use of evidence-based practice in nursing. *Journal of Advanced Nursing, 73*(8), 1910-1923. doi:<https://dx.doi.org/10.1111/jan.13280>

Thiel, L., & Ghosh, Y. (2008). Determining registered nurses' readiness for evidence-based practice. *Worldviews on Evidence-Based Nursing, 5*(4), 182-192. doi:<https://dx.doi.org/10.1111/j.1741-6787.2008.00137.x>

Toole, B. M., Stichler, J. F., Ecoff, L., & Kath, L. (2013). Promoting nurses' knowledge in evidence-based practice: do educational methods matter? *Journal for Nurses in Professional Development, 29*(4), 173-181. doi:<https://dx.doi.org/10.1097/NND.0b013e31829aebdf>

Upton, D., & Upton, P. (2006). Development of an evidence-based practice questionnaire for nurses. *Journal of Advanced Nursing, 53*(4), 454-458. doi:<https://dx.doi.org/10.1111/j.1365-2648.2006.03739.x>

Xie, H. T., Zhou, Z. Y., Xu, C. Q., Ong, S., & Govindasamy, A. (2015). Nurses' attitudes towards research and evidence-based practice. *Annals of the Academy of Medicine Singapore, 44*, S240.

Yip, W. K., Mordiffi, S. Z., Majid, M. S., & Ang, E. K. N. (2010). Nurses' perspective towards evidence-based practice: A descriptive study. *Annals of the Academy of Medicine Singapore, 39*, S372.
