# Supplementary material for: Development and content validation of a measure to assess evidence-informed decision-making competence in public health nursing
Source: PLoS One. 2021 Mar 10;16(3):e0248330. doi: 10.1371/journal.pone.0248330 (PMC7946311; doi:10.1371/journal.pone.0248330)
Supplement: S2 Table — (DOCX) [file pone.0248330.s002.docx]

**S2 Table. Detailed content coverage chart per EIDM attitudes/beliefs measure.**

| **Tool** | **EIDM Content Coverage** | | |
| --- | --- | --- | --- |
| n=17 measures total; n=15 measures with retrieved items | **General Beliefs about EIDM** | **Individual Factors** | **Organizational Factors** |
| **EBPQ (Upton & Upton, 2006)** |  |  |  |
| **SN-EBP (Adams, 2007)** |  |  |  |
| **Self-developed measure by Chiu et al. (2010)** |  |  |  |
| **Persian translated EBP measure (Seyyedrasooli, Zamanzadeh, Valizadeh, & Tadaion, 2012)** | *Unable to retrieve items. | | |
| **Self-developed measure by Yip, Mordiffi, Majid, and Ang (2010)** |  |  |  |
| **Self-developed measure by Chew, Sim, Sim, and Yan (2015)** | *Unable to retrieve items. | | |
| **Self-developed EBP measure by Melnyk et al. (2004)** |  |  |  |
| **Modified Evidence-Based Nursing Education Questionnaire (EBEQ) (Hellier & Cline, 2016)** |  |  |  |
| **Quick EBP VIK (Values, Implementation, Knowledge) Survey (Linda Connor, 2017; L. Connor, Paul, McCabe, & Ziniel, 2017)** |  |  |  |
| **Self-developed measure by Barako, Chege, Wakasiaka, and Omondi (2012)** |  |  |  |
| **EBP measure developed by by Majid et al. (2011) (Adamu & Naidoo, 2015; Farokhzadian, Khajouei, & Ahmadian, 2015)** |  |  |  |
| **Evidence-based Practice Beliefs Scale (Melnyk, Fineout-Overholt, & Mays, 2008)** |  |  |  |
| **Modified Korean Evidence-Based Medicine questionnaire (Park, Ahn, & Park, 2015)** |  |  |  |
| **Evidence-Based Practice Attitudes Scale (EBPAS) (Duffy, Culp, Sand-Jecklin, Stroupe, & Lucke-Wold, 2016; Duffy et al., 2015)** |  |  |  |
| **Attitudes to Evidence-Based Practice Questionnaire (Linton & Prasun, 2013)** |  |  |  |
| **Evidence-Based Nursing Attitude Questionnaire (EBNAQ) (Almaskari, 2017; Ruzafa-Martinez, Lopez-Iborra, & Madrigal-Torres, 2011)** |  |  |  |
| **Nurses’ Attitudes Toward EBP Scale (NATES) (Thiel & Ghosh, 2008)** |  |  |  |
| **Total # measures addressing each EIDM attitudes/beliefs domain** | **14** | **6** | **5** |

Adams, S. L. (2007). *Understanding the variables that influence translation of evidence-based practice into school nursing.* (Ph.D.), University of Iowa, Retrieved from <http://search.ebscohost.com/login.aspx?direct=true&db=cin20&AN=109853626&site=ehost-live> (Dissertation/Thesis)

Adamu, A., & Naidoo, J. R. (2015). Exploring the perceptions of registered nurses towards evidence-based practice in a selected general hospital in Nigeria. *Africa Journal of Nursing and Midwifery, 17*(1), 33-46.

Almaskari, M. (2017). *Omani staff nurses' and nurse leaders' attitudes toward and perceptions of barriers and facilitators to the implementation of evidence-based practice.* (10277864), Widener University, Ann Arbor. (Dissertation/Thesis)

Barako, T. D., Chege, M., Wakasiaka, S., & Omondi, L. (2012). Factors influencing application of evidence-based practice among nurses. *African Journal of Midwifery & Women's Health, 6*(2), 71-77.

Chew, M. L., Sim, K. H., Sim, Y. F., & Yan, C. C. (2015). Attitudes, skills and knowledge of primary healthcare nurses on the use of evidence-based nursing (EBN) and barriers influencing the use of EBN in the primary healthcare setting. *Annals of the Academy of Medicine Singapore, 1)*, S503.

Connor, L. (2017). *Pediatric nurses' knowledge, values, and implementation of evidence-based practice and use of two patient safety goals.* (10269833), University of Massachusetts Boston, Ann Arbor. (Dissertation/Thesis)

Connor, L., Paul, F., McCabe, M., & Ziniel, S. (2017). Measuring Nurses' Value, Implementation, and Knowledge of Evidence-Based Practice: Further Psychometric Testing of the Quick-EBP-VIK Survey. *Worldviews on Evidence-Based Nursing, 14*(1), 10-21. doi:<https://dx.doi.org/10.1111/wvn.12190>

Duffy, J. R., Culp, S., Sand-Jecklin, K., Stroupe, L., & Lucke-Wold, N. (2016). Nurses' Research Capacity, Use of Evidence, and Research Productivity in Acute Care: Year 1 Findings From a Partnership Study. *Journal of Nursing Administration, 46*(1), 12-17. doi:<https://dx.doi.org/10.1097/NNA.0000000000000287>

Duffy, J. R., Culp, S., Yarberry, C., Stroupe, L., Sand-Jecklin, K., & Sparks Coburn, A. (2015). Nurses' research capacity and use of evidence in acute care: baseline findings from a partnership study. *Journal of Nursing Administration, 45*(3), 158-164. doi:<https://dx.doi.org/10.1097/NNA.0000000000000176>

Farokhzadian, J., Khajouei, R., & Ahmadian, L. (2015). Evaluating factors associated with implementing evidence-based practice in nursing. *Journal of Evaluation in Clinical Practice, 21*(6), 1107-1113. doi:<https://dx.doi.org/10.1111/jep.12480>

Hellier, S., & Cline, T. (2016). Factors that affect nurse practitioners' implementation of evidence-based practice. *Journal of the American Association of Nurse Practitioners, 28*(11), 612-621. doi:<https://dx.doi.org/10.1002/2327-6924.12394>

Linton, M. J., & Prasun, M. A. (2013). Evidence-based practice: collaboration between education and nursing management. *Journal of Nursing Management, 21*(1), 5-16. doi:<https://dx.doi.org/10.1111/j.1365-2834.2012.01440.x>

Melnyk, B. M., Fineout-Overholt, E., & Mays, M. Z. (2008). The evidence-based practice beliefs and implementation scales: psychometric properties of two new instruments. *Worldviews on Evidence-Based Nursing, 5*(4), 208-216. doi:<https://dx.doi.org/10.1111/j.1741-6787.2008.00126.x>

Park, J. W., Ahn, J. A., & Park, M. M. (2015). Factors influencing evidence-based nursing utilization intention in Korean practice nurses. *International Journal of Nursing Practice, 21*(6), 868-875. doi:<https://dx.doi.org/10.1111/ijn.12296>

Ruzafa-Martinez, M., Lopez-Iborra, L., & Madrigal-Torres, M. (2011). Attitude towards Evidence-Based Nursing Questionnaire: development and psychometric testing in Spanish community nurses. *Journal of Evaluation in Clinical Practice, 17*(4), 664-670. doi:<https://dx.doi.org/10.1111/j.1365-2753.2011.01677.x>

Seyyedrasooli, A., Zamanzadeh, V., Valizadeh, L., & Tadaion, F. (2012). Individual Potentials Related to Evidence-Based Nursing among Nurses in Teaching Hospitals Affiliated to Tabriz University of Medical Sciences, Tabriz, Iran. *Journal of Caring Sciences, 1*(2), 93-99. doi:<https://dx.doi.org/10.5681/jcs.2012.014>

Thiel, L., & Ghosh, Y. (2008). Determining registered nurses' readiness for evidence-based practice. *Worldviews on Evidence-Based Nursing, 5*(4), 182-192. doi:<https://dx.doi.org/10.1111/j.1741-6787.2008.00137.x>

Upton, D., & Upton, P. (2006). Development of an evidence-based practice questionnaire for nurses. *Journal of Advanced Nursing, 53*(4), 454-458. doi:<https://dx.doi.org/10.1111/j.1365-2648.2006.03739.x>

Yip, W. K., Mordiffi, S. Z., Majid, M. S., & Ang, E. K. N. (2010). Nurses' perspective towards evidence-based practice: A descriptive study. *Annals of the Academy of Medicine Singapore, 39*, S372.
