# Supplementary material for: Development and content validation of a measure to assess evidence-informed decision-making competence in public health nursing
Source: PLoS One. 2021 Mar 10;16(3):e0248330. doi: 10.1371/journal.pone.0248330 (PMC7946311; doi:10.1371/journal.pone.0248330)
Supplement: S3 Table — (DOCX) [file pone.0248330.s003.docx]

**S3 Table. Response scales of measures.**

| **Tool** | **Objective** | | **Self-report** | | | |
| --- | --- | --- | --- | --- | --- | --- |
|  | **Multiple choice** | **Open text/Short answer** | **Agreement level**  (e.g., strongly disagree to strongly agree) | **Quality rating** (e.g. poor to best; not competent to highly competence) | **Frequency**  (e.g., never to frequently) | **Confidence level**  (e.g., I feel confident I can) |
| **Knowledge**  (n=19 measures total; n=16 measures with retrieved response type) | | | | | | |
| **Evidence-Based Practice Questionnaire (EBPQ) (Upton & Upton, 2006)** |  |  |  |  |  |  |
| **School Nursing Evidence-Based Practice Questionnaire (SN-EBP) (Adams, 2007)** |  |  |  |  |  |  |
| **Self-developed measure by Chiu et al. (2010)** |  | **)** |  |  |  |  |
| **Johns Hopkins Nursing EBP Assessment Survey (Bissett, Cvach, & White, 2016)** |  |  |  |  |  |  |
| **Persian translated EBP measure (Seyyedrasooli, Zamanzadeh, Valizadeh, & Tadaion, 2012)** | *Unable to retrieve response type. | | | | | |
| **Self-developed measure by Yip et al. (Yip, Mordiffi, Majid, & Ang, 2010)** |  |  |  |  |  |  |
| **Self-developed measure by Chew, Sim, Sim, and Yan (2015)** | *Unable to retrieve response type. | | | | | |
| **Self-developed EBP measure by Melnyk et al. (2004)** |  |  |  |  |  |  |
| **Modified Evidence-Based Nursing Education Questionnaire (EBEQ)**  **(Hellier & Cline, 2016)** |  |  |  |  |  |  |
| **Quick EBP VIK (Values, Implementation, Knowledge) Survey (Connor, 2017; Connor, Paul, McCabe, & Ziniel, 2017)** |  |  |  |  |  |  |
| **Modified Stevens EBP Readiness Inventory (ERI) (Finnish ERI) (Saunders, Stevens, & Vehvilainen-Julkunen, 2016)** |  |  |  |  |  |  |
| **Self-developed measure by Gerrish et al. (2011)** |  |  |  |  |  |  |
| **Knowledge and Skills in Evidence-Based Nursing (KS-EBN) (Gu, Ha, & Kim, 2015)** |  |  |  |  |  |  |
| **Adapted Fresno Test (Laibhen-Parkes, 2014)** |  |  |  |  |  |  |
| **Single item measure for EBP knowledge by Skela-Savic, Hvalic-Touzery, and Pesjak (2017)** |  |  |  |  |  |  |
| **Perceived EBP Knowledge Measure (Thiel & Ghosh, 2008)** |  |  |  |  |  |  |
| **Evidence-Based Practice Knowledge**  **Assessment in Nursing (EKAN) (Hagedorn Wonder et al., 2017)** |  |  |  |  |  |  |
| **Knowledge Assessment Test (KAT) (Xie, Zhou, Xu, Ong, & Govindasamy, 2015)** | *Unable to retrieve response type. | | | | | |
| **Core Knowledge Questionnaire (Toole, Stichler, Ecoff, & Kath, 2013)** |  |  |  |  |  |  |
| **Total # knowledge measures for each response type** | **4** | **3** | **5** | **5** | **0** | **1** |
| **Skills**  (n=15 measures; n=12 measures with retrieved response type) | | | | | | |
| **EBPQ (Upton & Upton, 2006)** |  |  |  |  |  |  |
| **SN-EBP (Adams, 2007)** |  |  |  |  |  |  |
| **Self-developed measure by Chiu et al. (2010)** |  |  |  |  |  |  |
| **Johns Hopkins Nursing EBP Assessment Survey (Bissett et al., 2016)** |  |  |  |  |  |  |
| **Persian translated EBP measure (Seyyedrasooli et al., 2012)** | *Unable to retrieve response type. |  |  |  |  |  |
| **Self-developed measure by Yip et al. (2010)** |  |  |  |  |  |  |
| **Self-developed measure by Chew et al. (2015)** | *Unable to retrieve response type. | | | | | |
| **EBP measure developed by Majid et al. (2011) (Adamu & Naidoo, 2015; Farokhzadian, Khajouei, & Ahmadian, 2015)** |  |  |  |  |  |  |
| **Modified Stevens EBP Readiness Inventory (ERI) (Finnish ERI) (Saunders et al., 2016)** |  |  |  |  |  |  |
| **Self-developed measure by Gerrish** **et al. (2011)** |  |  |  |  |  |  |
| **Knowledge and Skills in Evidence-Based Nursing (KS-EBN) (Gu et al., 2015)** |  |  |  |  |  |  |
| **Adapted Fresno Test (Laibhen-Parkes, 2014)** |  |  |  |  |  |  |
| **Self-developed measure by Gerrish and Clayton (2004)** |  |  |  |  |  |  |
| **DEBPQ (Gerrish et al., 2007)** |  |  |  |  |  |  |
| **Information literacy tool (Sim, Jang, & Kim, 2016)** | *Unable to retrieve response type. | | | | | |
| **Total # skills measures for each response type** | **1** | **2** | **3** | **6** | **0** | **2** |
| **Behaviours**  (n=13 measures total and with retrieved response type) | | | | | | |
| **EBPQ (Upton & Upton, 2006)** |  |  |  |  |  |  |
| **SN-EBP (Adams, 2007)** |  |  |  |  |  |  |
| **Self-developed measure by Chiu et al. (2010)** |  |  |  |  |  |  |
| **Johns Hopkins Nursing EBP Assessment Survey (Bissett et al., 2016)** |  |  |  |  |  |  |
| **Self-developed EBP measure by Melnyk et al. (2004)** |  |  |  |  |  |  |
| **Modified Evidence-Based Nursing Education Questionnaire (EBEQ) (Hellier & Cline, 2016)** |  |  |  |  |  |  |
| **Quick EBP VIK (Values, Implementation, Knowledge) Survey (Connor, 2017; Connor et al., 2017)** |  |  |  |  |  |  |
| **Self-developed measure by Barako, Chege, Wakasiaka, and Omondi (2012)** |  |  |  |  |  |  |
| **EBP Implementation Scale (Melnyk, Fineout-Overholt, & Mays, 2008)** |  |  |  |  |  |  |
| **Self-developed measure by Bostrom, Rudman, Ehrenberg, Gustavsson, and Wallin (2013)** |  |  |  |  |  |  |
| **Self-developed measure by (Kim et al., 2013)** |  |  |  |  |  |  |
| **Evidence-Based Practice Confidence Scale (EPIC) (Duffy, Culp, Sand-Jecklin, Stroupe, & Lucke-Wold, 2016; Duffy et al., 2015)** |  |  |  |  |  |  |
| **EBP Competency Tool (Melnyk et al., 2018)** |  |  |  |  |  |  |
| **Total # behaviour measures addressing each EIDM behaviours domain** | **0** | **0** | **2** | **2** | **8** | **2** |
