# Supplementary material for: Development and content validation of a measure to assess evidence-informed decision-making competence in public health nursing
Source: PLoS One. 2021 Mar 10;16(3):e0248330. doi: 10.1371/journal.pone.0248330 (PMC7946311; doi:10.1371/journal.pone.0248330)
Supplement: S4 Table — (DOCX) [file pone.0248330.s004.docx]

**S4 Table. Item content validity indexes (CVI).**

| **Original Items – EIDM Knowledge** | **Item CVI** |
| --- | --- |
| 1. Knowledge of what is involved in the ‘define’ step of EIDM. | 1.00 |
| 1. Knowledge of what is involved in the ‘search’ step of EIDM. | 1.00 |
| 1. Knowledge of what is involved in the ‘appraise’ step of EIDM. | 1.00 |
| 1. Knowledge of what is involved in the ‘synthesize’ step of EIDM. | 1.00 |
| 1. Knowledge of what is involved in the ‘adapt’ step of EIDM. | 1.00 |
| 1. Knowledge of what is involved in the ‘implement’ step of EIDM. | 1.00 |
| 1. Knowledge of what is involved in the ‘evaluate’ step of EIDM. | 1.00 |
| 1. Knowledge about the 6S hierarchy of research evidence | 0.82 |
| 1. Knowledge of online databases that house pre-appraised, synthesized research evidence (e.g., Health Evidence, ACCESSSS) | 0.82 |
| 1. Knowledge of online databases that house individual research studies (e.g., Medline) | 0.91 |
| 1. Knowledge of critical appraisal tools for summaries of research evidence (e.g. clinical practice guidelines) | 0.91 |
| 1. Knowledge of critical appraisal tools for systematic reviews | 0.91 |
| 1. Knowledge of critical appraisal tools for qualitative research studies | 0.82 |
| 1. Knowledge of critical appraisal tools for randomized controlled trials | 0.91 |
| 1. Knowledge of critical appraisal tools for observational studies | 0.82 |
| 1. Knowledge about the definition of knowledge translation | 0.73 |
| 1. Knowledge of **all** the steps of developing a knowledge translation plan (conduct stakeholder analysis, assess barriers/facilitators, select appropriate knowledge translation strategies) | 0.82 |
| 1. Knowledge about how to develop outcome indicators to evaluate practice change. | 0.72 |
| 1. Knowledge about how to develop process indicators to evaluate practice change. | 0.72 |
| **Original Items – EIDM Skills** | **Item CVI** |
| 1. Ability to develop an answerable public health practice question using the PICO (P=population; I=intervention; C=comparison; O=outcome) format for quantitative research questions. | 1.00 |
| 1. Ability to develop an answerable public health practice question using the PS (P=patient/population; S=situation) format for qualitative research questions. | 0.91 |
| 1. Ability to develop a comprehensive strategy to search for research evidence. | 0.91 |
| 1. Ability to use online databases that house pre-appraised, synthesized research evidence (e.g., Health Evidence). | 0.91 |
| 1. Ability to use online databases that house individual research studies reports (e.g., CINAHL) | 0.91 |
| 1. Ability to use critical appraisal tools to appraise pre-appraised, synthesized research evidence such as best practice guidelines. | 0.91 |
| 1. Ability to use critical appraisal tools to appraise systematic reviews | 0.82 |
| 1. Ability to use critical appraisal tools to appraise qualitative research studies | 0.82 |
| 1. Ability to use critical appraisal tools to appraise randomized controlled trials | 0.91 |
| 1. Ability to use critical appraisal tools to appraise observational studies | 0.82 |
| 1. Ability to assess the applicability of research evidence to the local public health context. | 1.0 |
| 1. Ability to develop a knowledge translation plan for implementing a new change in practice. | 0.91 |
| 1. Ability to develop a knowledge translation plan for de-implementing a current practice. | 0.91 |
| 1. Ability to develop outcome indicators to evaluate practice change. | 0.72 |
| 1. Ability to develop process indicators to evaluate practice change. | 0.72 |
| **Original Items – EIDM Attitudes/Beliefs^1^** | **Item CVI** |
| 1. I am sure that I can implement EBP in a time efficient way. | 0.82 |
| 1. I am sure that I can implement EBP. | 0.72 |
| 1. I believe that I can search for the best evidence to answer clinical questions in a time efficient way. | 0.91 |
| 1. I am confident about my ability to implement EBP where I work. | 0.72 |
| 1. I believe that I can overcome barriers in implementing EBP. | 0.91 |
| 1. I am sure about how to measure the outcomes of clinical care. | 0.91 |
| 1. I know how to implement EBP sufficiently enough to make practice changes. | 0.72 |
| 1. I am sure that I can access the best resources in order to implement EBP. | 0.82 |
| 1. I am sure that implementing EBP will improve the care that I deliver to my patients. | 0.82 |
| 1. I believe that critically appraising evidence is an important step in the process. | 0.91 |
| 1. I am clear about the steps of EBP. | 0.64 |
| 1. I am sure that evidence-based guidelines can improve clinical care. | 0.82 |
| 1. I believe that EBP results in the best clinical care for patients. | 0.64 |
| 1. I believe the care that I deliver is evidence-based. | 0.72 |
| 1. I believe EBP is difficult. (reverse scored) | 0.82 |
| 1. I believe that EBP takes too much time. (reverse scored) | 0.72 |
| **Original Items – EIDM Behaviours^2^** | **Item CVI** |
| 1. Questions clinical practices for the purpose of improving the quality of care. | 0.91 |
| 1. Describes clinical problems using internal evidence* (internal evidence* = evidence generated internally within a clinical setting, such as patient assessment data, outcomes management, and quality improvement data). | 0.91 |
| 1. Participates in the formulation of clinical questions using PICOT* format. (*PICOT = patient; population; intervention or area of interest; comparison intervention or group; outcome; time). | 0.91 |
| 1. Searches for external evidence* to answer focused clinical questions. (external evidence* = evidence generated from research). | 0.91 |
| 1. Participates in critical appraisal of pre-appraised evidence (such as clinical practice guidelines, evidence-based policies and procedures, and evidence syntheses). | 0.82 |
| 1. Participates in the critical appraisal of published research studies to determine their strength and applicability to clinical practice. | 0.91 |
| 1. Participates in the evaluation and synthesis of a body of evidence gathered to determine its’ strength and applicability to clinical practice. | 0.91 |
| 1. Collects practice data (e.g., individual patient data, quality improvement data) systematically as internal evidence for clinical decision making in the care of individuals, groups and populations. | 0.72 |
| 1. Integrates evidence gathered from external and internal sources in order to plan evidence-based practice changes. | 0.91 |
| 1. Implements practice changes based on evidence and clinical expertise and patient preferences to improve care processes and patient outcomes. | 0.82 |
| 1. Evaluates outcomes of evidence-based decisions and practice changes for individuals, groups and populations to determine best practices. | 0.91 |
| 1. Disseminates best practices supported by evidence to improve quality of care and patient outcomes. | 0.82 |
| 1. Participates in strategies to sustain an evidence-based practice culture. | 0.91 |

1. Melnyk, B. M., Fineout-Overholt, E., & Mays, M. Z. (2008). The evidence-based practice beliefs and implementation scales: psychometric properties of two new instruments. *Worldviews on Evidence-Based Nursing, 5*(4), 208-216. doi:https://dx.doi.org/10.1111/j.1741- 6787.2008.00126.x

2. Melnyk, B. M., Gallagher‐Ford, L., Zellefrow, C., Tucker, S., Thomas, B., Sinnott, L. T., & Tan, A. (2018). The First U.S. Study on Nurses’ Evidence Based Practice Competencies Indicates Major Deficits That Threaten Healthcare Quality, Safety, and Patient Outcomes. *Worldviews on Evidence-Based Nursing, 15*(1), 16-25. doi:10.1111/wvn.12269
